# North American experience with Low protein diet for Non-dialysis-dependent chronic kidney disease

**DOI:** 10.1186/s12882-016-0304-9

**Published:** 2016-07-19

**Authors:** Kamyar Kalantar-Zadeh, Linda W. Moore, Amanda R. Tortorici, Jason A. Chou, David E. St-Jules, Arianna Aoun, Vanessa Rojas-Bautista, Annelle K. Tschida, Connie M. Rhee, Anuja A. Shah, Susan Crowley, Joseph A. Vassalotti, Csaba P. Kovesdy

**Affiliations:** Harold Simmons Center for Kidney Disease Research and Epidemiology, Division of Nephrology & Hypertension, University of California Irvine Medical Center, 101 The City Drive South, Orange, CA 92868-3217 USA; Long Beach Veterans Affairs Healthcare System, Long Beach, CA USA; Department Epidemiology, UCLA Fielding School of Public Health, University of California Los Angeles, Los Angeles, CA USA; Los Angeles Biomedical Research Institute at Harbor-UCLA Medical Center, Torrance, CA USA; Houston Methodist Hospital, Houston, TX USA; Center for Healthful Behavior Change, Department of Population Health, New York University School of Medicine, New York, NY USA; Louis Stokes Cleveland VA Medical Center, Cleveland, OH USA; VA Connecticut Healthcare System, West Haven, CT USA; Icahn School of Medicine at Mount Sinai, New York, NY USA; National Kidney Foundation, Inc., New York, NY USA; University of Tennessee Health Science Center, Memphis, TN USA

**Keywords:** Dietary restriction, Dietary protein intake, Low protein diet, Nutritional management, CKD

## Abstract

Whereas in many parts of the world a low protein diet (LPD, 0.6-0.8 g/kg/day) is routinely prescribed for the management of patients with non-dialysis-dependent chronic kidney disease (CKD), this practice is infrequent in North America. The historical underpinnings related to LPD in the USA including the non-conclusive results of the *Modification of Diet in Renal Disease* Study may have played a role. Overall trends to initiate dialysis earlier in the course of CKD in the US allowed less time for LPD prescription. The usual dietary intake in the US includes high dietary protein content, which is in sharp contradistinction to that of a LPD. The fear of engendering or worsening protein-energy wasting may be an important handicap as suggested by a pilot survey of US nephrologists; nevertheless, there is also potential interest and enthusiasm in gaining further insight regarding LPD’s utility in both research and in practice. Racial/ethnic disparities in the US and patients’ adherence are additional challenges. Adherence should be monitored by well-trained dietitians by means of both dietary assessment techniques and 24-h urine collections to estimate dietary protein intake using urinary urea nitrogen (UUN). While keto-analogues are not currently available in the USA, there are other oral nutritional supplements for the provision of high-biologic-value proteins along with dietary energy intake of 30–35 Cal/kg/day available. Different treatment strategies related to dietary intake may help circumvent the protein- energy wasting apprehension and offer novel conservative approaches for CKD management in North America.

**Electronic supplementary material:**

The online version of this article (doi:10.1186/s12882-016-0304-9) contains supplementary material, which is available to authorized users.

## Why a low protein diet is not well received in the USA

Prescribing and reinforcing a low protein diet (LPD) as a means of conservatively managing chronic kidney disease (CKD) progression is not largely practiced in North America. This may be due to a variety of reasons including lack of strong evidence about the efficacy and safety of such dietary approaches, especially in populations with preexisting nutritional challenges such as the elderly and those with diabetes, which represent a growing proportion of patients with CKD. Additionally, the inconclusive results from a large US randomized trial in the early 1990’s, the *Modification of Diet in Renal Disease* (MDRD) study [1], played a key role to this end. Another likely reason is that many nephrologists in the USA and Canada lack the needed education, insight, and prior training and experience related to LPD, while they are exceptionally well trained to prepare CKD patients for the transition to conventional (full-dose) dialysis treatment. The rise of the dialysis industry and recommendations by nephrology guidelines to initiate dialysis earlier rather than later have each contributed to these trends over the past two decades. Fear of what used to be called “malnutrition,” now referred to as the PEW or “malnutrition-inflammation cachexia syndrome,” is another potential barrier, especially since among the many various complications of CKD those affecting nutritional status including both loss of structural (muscle mass loss) and visceral proteins (hypoalbuminemia) are among the strongest predictors of poor outcomes [2, 3].

Given the above conditions and concerns, the question remains as to why should we revisit the use of LPD for the conservative management of CKD in North America. A recent trial suggested that there was no survival benefit in starting patients earlier on dialysis [4]. This landmark study, supported by an increasing number of observational data in recent years, have paved the way for revisiting more conservative approaches in managing patients with advanced CKD who may prefer to avoid dialysis as long as possible [5]. Nevertheless, the concern about PEW along with inadequate training for the conservative management of CKD are two important barriers for bringing the LPD back to North America.

## Dietary protein intake in Americans with and without CKD

The designation of the LPD that is recommended for the management of CKD refers to a dietary protein intake of 0.6 to 0.8 grams of protein per kilogram of ideal body weight per day (g/kg/day). This amount is currently much lower than what is consumed in the USA. It is important to note that according to the *Food and Nutrition Board* (FNB) of the National Academy of Sciences of the USA, the *minimum* dietary protein requirement of a normal healthy (non-pregnant, non-lactating) adult person is indeed 0.6 g/kg/day; however, the FNB has stipulated adding a 33 % safety margin to this minimum amount, so that the “Recommended Dietary Allowance” (RDA) for healthy adults is to be 0.8 g/kg/day [6]. Hence, because a diet restricted to 0.6 g/kg/day protein intake is 25 % below the recommended 0.8 g/kg/day, the term “LPD” has generally been used to describe it, as it can be argued that giving 25 % less protein on a long-term basis might be inadequate for certain periods of time where the body is in an anabolic state (e.g. recovery from illnesses or injuries) [6]. For a stable (e.g. non-nephrotic, non-inflamed/non-catabolic) NDD-CKD patient, the so-called LPD of 0.60–0.80 g/kg/day represents sufficient protein intake—especially because the diet prescription includes the stipulation that 50 % of the protein should be of high biological value (see below).

**Fig. 1:**
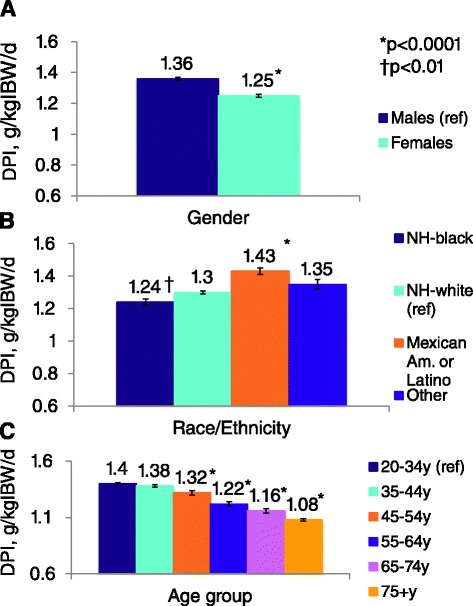
Estimated DPI in the USA across gender, race, and age; normalized to protein in g/kgIBW/d, for adults in the USA depicted for (**a**) sex, (**b**) race or ethnicity, and (**c**) age group. Analysis of variance for each panel, *p*<0.0001. Per panel, pairwise comparisons with each reference (ref) group: *p<0.0001, †*p*<0.01. Adapted from secondary NHANES data analyses by Moore et al. (with permission) [7]

According to a recent study by Moore et al. [7] who examined the pattern of dietary protein intake in the general population in the USA, an average American currently eats 1.3 g/kg/day. In this study the investigators examined dietary data from 16,872 adults (>20 years) who had participated in a contemporary phase of the National Health and Nutrition Examination Survey (NHANES) in the USA between 2001 and 2008 and who had completed a dietary interview and a 24-h diet recall [7]. According to these data, US women and men eat on average 1.25 and 1.36 g/kg/day of protein, respectively (see Fig. 1). Across race and ethnicity, Hispanics reported the highest dietary protein intake of 1.43 g/kg/day, while blacks exhibited the lowest amount at 1.24 g/kg/day. Dietary protein intake declined across advancing categories of age (see Fig. 1), but notably was still >1 g/kg/day even for those over 75 years of age.

These data should be juxta-positioned to a typical LPD of 0.6 to 0.8 g/kg/day. Indeed the strict form of LPD is expected to have a protein content closer to 0.6 g/kg/day with only less than +/−0.1 g/kg/day variation [7]. At least half of this amount should be of high biologic value (HBV) protein, such as those provided by animal and dairy products, to assure provision of essential amino acids. The “biological value” (BV) is the ratio of nitrogen incorporated into the body over nitrogen absorbed. BV is different from absorbability or bioavailability (i.e., how readily the protein can be digested and absorbed by the intestinal tract). Amino acid composition is the most important factor, as essential amino acids (EAA) missing from the diet prevent the synthesis of proteins that require them. If a protein source is missing EAAs, then its biological value will be low, as the missing EAAs form a bottleneck in protein synthesis (see Table 1).

**Table 1:** Biologic value of selected protein-rich foods. On a scale with 100 representing the highest efficiency. Foods with high biologic value (HBV) need to have a total score >75 (Source: Wikidoc on line: www.wikidoc.org/index.php/Biological_value)

	Ile	Leu	Val	Thr	Met & Cys	Trp	Lys	Phe & Tyr	His	Biologic value
Whole egg	1	1	1	1	1	1	1	1	1	95
Milk, human	1.1	1.4	1	1	1.1	1.6	1	1	0.9	95
Milk, cow	1.1	1.3	1	0.9	0.7^a^	1.3	1.3	0.9	1.1	90
Muscle, beef	0.8	0.9	0.7	0.9	0.9	0.9	1.4	0.7^a^	1.6	76
Soybeans	1.0	0.9	0.8	0.8	0.6^a^	1.3	1.1	1.0	1.4	75^a^
Rice	0.8	0.9	0.9	0.8	0.9	1.2	0.5^a^	1.2	0.8	75^a^
Wheat	0.6	0.8	0.6	0.7	0.8	1.1	0.4	0.8	1	67^a^
Potatoes	0.6	1.1	0.8	1.3	0.6	1.9	1.4	0.8	1.1	67^a^
Oats	0.8	0.8	0.8	0.7^a^	0.6^a^	1.2	0.6^a^	1	1.1	66^a^
Corn	1	1.7	0.8	0.7^a^	1.1	0.5^a^	0.4^a^	1	1	60^a^

Amino-acid abbreviations: *Ile* Isoleucine, *Leu* Leucine, *Thr* Threonine, *Met* Methionine, *Cys* Cysteine, *Trp* Tryptophan, *Lys* Lysine, *Phe* Phenylalanine, *Tyr* Tyrosine, *His* Histidine

^a^indicate low biologic value

**Fig. 2:**
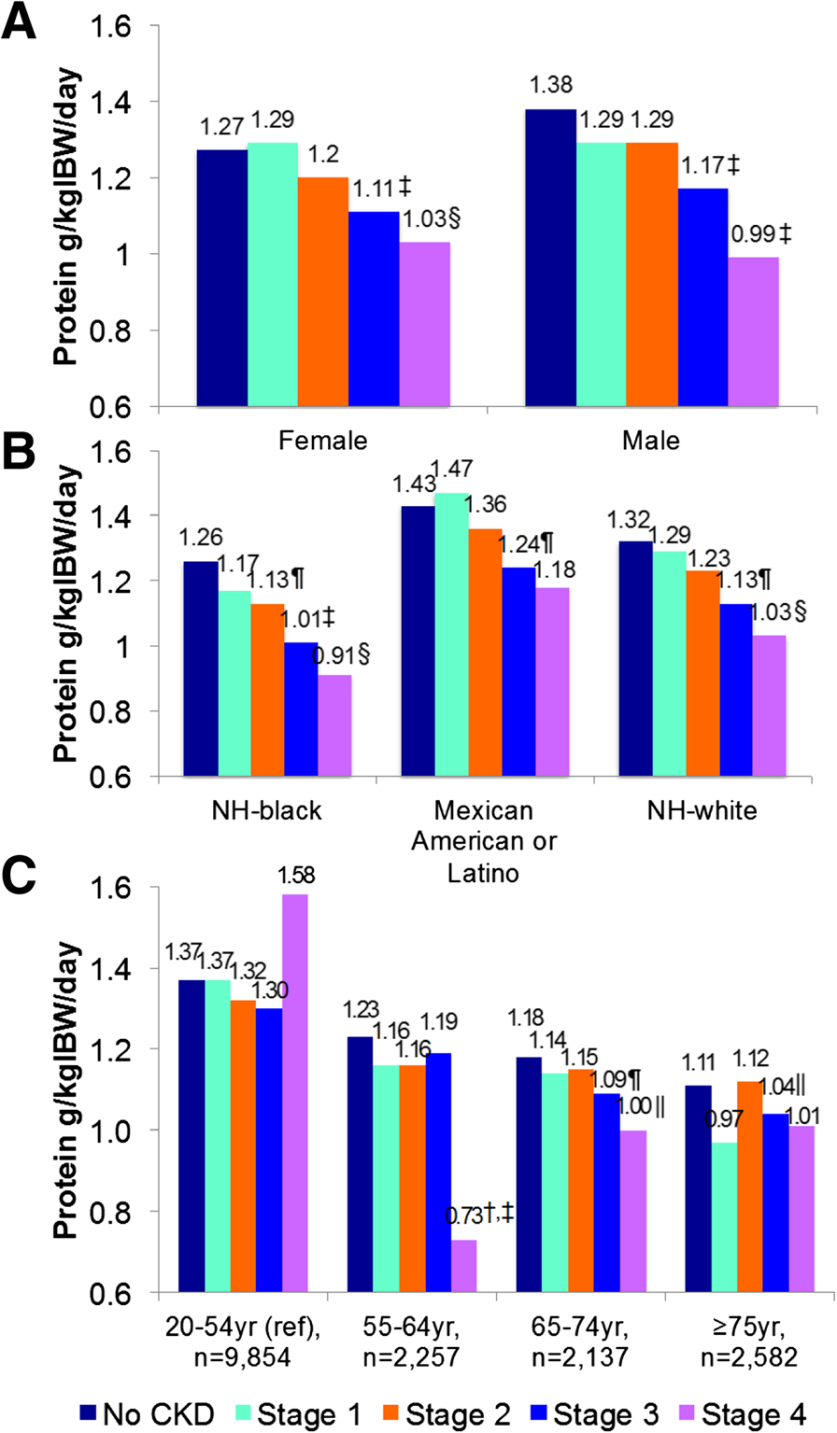
Estimated DPI in the USA across gender, race, and age accounting for stages of CKD: normalized to protein in g/kgIBW/d, for adults in the USA depicted for (**a**) sex, (**b**) race or ethnicity, and (**c**) age group. No evidence of CKD (No CKD), stage 1 CKD (eGFR, ≥90ml/min with kidney damage), stage 2 CKD (eGFR 60–89ml/min with kidney damage), stage 3 CKD (eGFR 30–59ml/min), or stage 4 CKD (eGFR <30 ml/min without dialysis). Overall FANOVA for each panel, *P*<0.0001. Per panel, pairwise comparisons with each reference group: *P<0.0001, †*P*<0.05. Per panel, pairwise comparisons with each subgroup ((**a**) females at each stage of CKD compared with NoCKD and males at each stage of CKD compared with NoCKD; (**b**) NH black at each stage of CKD compared with NoCKD, Mexican American or Latino at each stage of CKD compared with NoCKD, and NH white at each stage of CKD compared with NoCKD; (**c**) 20–54-year-olds at each stage of CKD compared with NoCKD, 55–64-year-olds at each stage of CKD compared with NoCKD, 65–74-year-olds at each stage of CKD compared with NoCKD, and 75+-year-olds at each stage of CKD compared with NoCKD): ‡*P*<0.0001, §*P*<0.001, ||*P*<0.01, ¶*P*<0.05. Adapted from secondary NHANES data analyses by Moore et al. (with permission) [7]

Given the above data, the average US citizen consumes a diet that is twice the amount of protein recommended for the management of CKD. It is important to note that with progressive decline in glomerular filtration rate (GFR), an unconscious decrease in dietary protein and energy intake is often observed in CKD patients [8]. This dietary decline is considered to be the result of the natural progression of CKD, especially when the estimated GFR (eGFR) falls below 25 ml/min/1.73 m^2^; hence, it is feared that such a decline in dietary protein intake may be accompanied by worsening nutritional status (see below) [9]. Although, most patients with CKD consume less dietary protein, the analyses of the NHANES data by Moore et al. [7] showed that after adjusting for age, the mean dietary protein intake of participants with CKD in the US was still high at 1.30 g/kg/day and did not differ between CKD Stages 1 and 2, i.e., 1.28 and 1.25 g/kg/day, respectively. Furthermore, although dietary protein intake was significantly different in Stages 3 and 4, i.e., 1.22 and 1.13 g/kg/day, respectively (see Fig. 2), it still remained well above the aforementioned LPD of 0.6 to 0.8 g/kg/day for Stages 3 and 4 CKD.

## Risk of protein energy wasting from LPD

An important challenge for reinvigorating enthusiasm for the LPD in the USA is the fear of PEW. There is little doubt that PEW is more likely to occur in later stages of CKD, especially when the eGFR is <25 ml/min/1.73 m^2^. To differentiate between various causes and consequences of the wasting syndrome in CKD, it is important to systematically define what is meant under the older designation of “protein-energy malnutrition.” [10] A working definition was advanced by Kalantar-Zadeh et al. in [11] as “the state of decreased body pools of protein with or without fat depletion or a state of diminished functional capacity, caused at least partly by inadequate nutrient intake relative to nutrient demand and/or which is improved by nutritional repletion.” We believe that this definition is applicable across all stages of CKD and encompasses what is also more recently referred to as PEW [11]. Hence, uremic malnutrition or wasting is engendered when the body’s need for protein and/or energy fuels cannot be satisfied by usual dietary intake.

Different conditions may contribute to PEW in CKD patients as discussed elsewhere [12]. In a recent study of 1,220 non-dialysis CKD patients by Kovesdy et al. [13], 45 % of participants had a serum albumin <3.8 g/dL and 22 % of subjects had a level <3.4 g/dL. Furthermore, the probability of PEW (defined as the presence of two or more out of three biochemical markers of PEW) showed a significant and linear increase with lower levels of eGFR [13]. In another study by Lawson et al. [14] in 50 CKD patients with serum creatinine >1.7 mg/dL, it was demonstrated that 20 % of patients were mildly-to-moderately malnourished, and 8 % were severely malnourished. Other similar studies including an analysis by Molnar et al. using the “malnutrition-inflammation score” have reported similar prevalences [15].

## Adjusting and monitoring dietary protein intake in CKD

Various renal nutrition guidelines [16, 17] recommend the achievement of certain thresholds of dietary protein intake in patients with moderate to advanced CKD with the goals of preventing the development of PEW or treating established PEW. The so-called “low” (LPD) and “very low protein diets” (VLPD) represent a daily protein intake of ~0.6 and ~0.3 g/kg/day [6]. Whereas the latter is very difficult to achieve and may be more likely associated with PEW risk, the former appears more practical and less risky. Many practicing nephrologists recommend a daily protein intake of 0.6 to 0.8 g/kg/day and monitor the adherence by estimating it (eDPI) using 24-h urinary urea nitrogen (UUN) where 1 g UN represents 6.25 g of protein and non-urea nitrogen excretion of 30 mg/kg/day [18] along with urinary protein losses if >5 g/day:

$$ \mathrm{eDPI}\ \left(\mathrm{g}/\mathrm{day}\right)=6.25*\mathrm{U}\mathrm{U}\mathrm{N}\ \left(\mathrm{g}/\mathrm{day}\right)+0.03*\mathrm{weight}\ \left(\mathrm{K}\mathrm{g}\right)+\mathrm{proteinuria}\ \left(\mathrm{g}/\mathrm{day}\right) $$

The same 24-h urine collection should also be used to examine 24-h urinary creatinine (to estimate muscle mass and to calculate 24-h creatinine clearance), potassium (target <2-3 g/day), phosphorus (target <1,000 mg/day), sodium (target <1.5-2 g/day although some coauthors suggest <3 g/day) and fluid intake (<1-1.5 liters/day). If the eDPI is >0.8 g/kg/day, more dietary counseling including higher intake of vegetarian meals may be considered. If the intake is <0.6 g/kg/day or if there are signs of PEW, oral nutritional supplementation with products specially designed for CKD should be considered. Because adequate calorie intake is needed to spare protein and prevent vitamin and mineral deficiencies, it is important to assure a dietary energy intake of at least 30–35 Cal/kg/day to avoid energy malnutrition.

In addition, it is important to assure provision of essential amino acids in form of HBV proteins, which include almost all animal protein sources (e.g., meats and dairy), and select plants (e.g., soybeans, quinoa) (Table 1). Note that this may be less protein than might be expected. For example, a 70-kg patient would require approximately 21 g/day of HBV protein (0.3 g/kg/day), which can be met by a single 3-oz serving of meat (about the size of a deck of cards). Protein supplementation by a variety of different methods has been shown to be effective in improving markers of PEW (for an exhaustive review see reference number [19]), and given the very robust association of these markers with poor outcomes, a strong case can be made in favor of such nutritional interventions. Importantly, optimal dietary protein can usually correct PEW irrespective of its etiology, as is the case for the strong argument behind the provision of nutritional support in cancer cachexia that is often engendered as a result of malignant disease and its associated pro-inflammatory cytokines and much less due to inadequate nutrition [20, 21].

A spontaneous reduction in protein intake as a result of anorexia in patients with reduced kidney function can be considered an adaptive process meant to alleviate the short-term consequences of the body’s inability to handle protein breakdown products [6]. The MDRD study detected small, but significant decreases in weight and serum concentrations of albumin, transferrin, and cholesterol associated with protein restriction; these were alluded to, but were not presented in detail in the original publication [22]. Furthermore, a study examining the incidence of ESRD and mortality after long-term observational follow-up of patients enrolled in MDRD study 2 (LPD vs. VLPD) indicated no significant difference in the incidence of ESRD, but a significantly higher mortality rate [adjusted hazard ratio (95 % CI): 1.92 (1.15 to 3.20)] in patients who had been randomized to the VLPD [23]. Due to such concerns, the focus has shifted to strategies designed to implement moderate protein restriction along with other measures to offset any effect towards PEW while maintaining its putative benefits.

## Attitudes of American practitioners towards a low protein diet

There are currently no data regarding the North American nephrologists’ and dietitians’ attitudes and perceptions of a LPD, although anecdotal reports suggest that the LPD is poorly received in the USA. This is despite the fact that multiple guidelines such as the Kidney Disease Outcome Quality Initiative (KDOQI) guidelines regarding dietary protein intake in diabetic kidney disease, developed by the National Kidney Foundation (NKF) of the USA in 2007 [24], suggests a dietary protein intake of 0.8 g/kg/day (see Table 2). A low protein intake is also a sustained recommendation of the national US Veterans Affairs (VA) and Department of Defense (DoD) clinical practice guideline for the management of patients with chronic kidney disease in primary care. (available at http://www.healthquality.va.gov/guidelines/CD/ckd/VADoDCKDCPG.pdf) Lastly, this is the same dietary protein intake recommended for all Americans by the US dietary guidelines. Attitudes and perceptions of LPD among clinicians may also differ for patients across racial and ethnic groups, contributing to differences observed in the general population protein intake from NHANES (see Fig. 1). The Academy of Nutrition and Dietetics Evidence Analysis Library recommends similar protein restrictions in its evidence-based CKD Medical Nutrition Therapy guidelines published in 2010 [17].

**Table 2:** Excerpts of the Kidney Disease Outcome Quality Initiative (KDOQI) guidelines regarding dietary protein intake in diabetic kidney disease, developed by the by the National Kidney Foundation (NKF) of the USA 2007 [24]

KDOQI Clinical Practice Guidelines and Clinical Practice Recommendations for Diabetes and Chronic Kidney Disease, 2007 GUIDELINE 5: NUTRITIONAL MANAGEMENT IN DIABETES AND CHRONIC KIDNEY DISEASE
Management of diabetes and CKD should include nutritional intervention. Dietary modifications may reduce progression of CKD. 5.1 Target dietary protein intake for people with diabetes and CKD stages 1–4 should be the RDA of 0.8 g/kg body weight per day. (B) BACKGROUND Nutritional management for people with diabetes has traditionally focused on blood glucose control. However, dietary protein intake at all stages of CKD appears to have an important impact in this population. If dietary protein is limited, adequate caloric intake must be maintained by increasing calories from carbohydrates and/or fats. Competing needs for nutritional management of hyperglycemia, hypertension, and dyslipidemia can make determination of appropriate protein intake challenging. Furthermore, the diet for diabetes and CKD should consider the qualitative, as well as the quantitative, aspects of proteins, carbohydrates, and fats. To address dietary recommendations for people with diabetes and CKD stages 1 to 4, studies evaluating interventions that reduced or altered sources of dietary protein and other nutrients were reviewed. Dietary recommendations for CKD stage 5 are provided in the KDOQI™ CPGs for Nutrition in Chronic Renal Failure. RATIONALE *A dietary protein intake of 0.8 g/kg body weight per day, the RDA for this macronutrient, is a level that has been achieved in studies of diabetes and CKD. Reduction in albuminuria and stabilization of kidney function have been reported with dietary protein intake at the RDA level. Nutrition surveys indicate that most people eat in excess of the RDA for dietary protein. (Moderate)*

We recently embarked on an online survey for nephrologists practicing in Veterans Administration medical centers. Based on responses from 16 nephrologists, 69 % of these physicians confirmed that they never or rarely recommended LPD to their patients with CKD (see Table 3). However, 81 % of nephrologists appeared to be interested in learning more about this therapy. In this survey, 44 % of the surveyed physicians suggested that the dedicated assignment of a dietitian to their CKD patient population is needed. The following are selected comments in response to the survey question, “How do you suggest nephrologists can help more effectively implement Low Protein Diet (LPD) protocols for management of CKD patients?” (1) “We would need additional help and support from the clinical nutritionists.” (2) “Dedicated renal dietitian … able to provide monthly follow-up… lack of dietitian is reason this is difficult to implement ….” (3) “Would need amino acid supplementation available in the US” (4) “… patients have many adherence issues and hard enough to get them to restrict potassium and very tough to get them to do LPD.” (5) “Identifying patients based on their clinical morbidities and working closely with nutrition services…” (6) “Scheduled follow up with nutrition expert and educating patients to maintain food diary…” (7) “Monitoring protein intake by 24-h urine collection…” (8) “Biggest barrier is dietitian involvement for education, teaching, and follow up monitoring…” (9) “Not useful except in highly motivated patients.” (10) “It’s really difficult for our patients to follow a low-protein diet.” (11) “MDRD study was essentially negative.” (12) “… patients are already malnourished near stage 5 and would not want to further impair their nutritional status.” (13) “… however, low protein diet may be important for a subset of CKD patients.” (14) “Since there is limited data on the efficacy of these diets in slowing progression – though may delay uremia – … first priority is adherence to medication and then if patient is able/willing … refer for LPD if not malnourished.” (15) “Limiting protein with monitoring for signs of malnutrition…” (16) “… the benefits of a low protein diet in slowing progression of CKD is minor particularly in more advanced CKD… would worry that the patients may become malnourished unless … followed very closely.” (17) “Anorexia and malnutrition are a big issue… cutting back can deeply malnourish a patient.” (18) “For diabetics, this is another impossible dietary imposition for minimal benefit.” (19) “Unclear if patients are actually adherent to the diet … think of how many are adherent to a low sodium diet…. too difficult to implement.” (20) “… mention to patients that it may work and … refer them to dietitian … <5 % are interested.” (21) “Maybe for the nephrotic syndrome patients.” (22) “To control uremia, … [low protein] diet only and it helped avoid [serum urea nitrogen] >100 mg/dL.” (23) “… patients did not fare well.” (24) “… tell patients to limit portion size and red meat as part of general health. … not convinced that LPD is useful outside of MDRD type intervention with lots of dietitian follow up.” (see Table 3 for summary of data).

**Table 3:** Pilot survey of nephrologists from the Veterans Administration health system (based on 16 preliminary sets of responses)

Question 1: Do you recommend or practice LPD?		Question 2: Will you be interested in implementing and managing LPD?		Question 3: How to suggest implement LPD more effectively?	
Never	13 %	No	25 %	Dedicated dietitian involvement needed	44 %
Rarely	56 %	Maybe	56 %	Need to improve patient adherence and education	19 %
Sometimes	25 %	Yes	19 %	Monitor protein intake including by 24-h urine	19 %
Frequently	6 %			Do not favor LPD	13 %
				Prioritize amino-acid and other supplements	6 %

Exact questions that were asked: Question 1: Do you recommend or implement Low Protein Diet (LPD) for conservative management of patients with moderate to advanced CKD, e.g. limiting daily dietary protein intake to 0.6-0.8 gram/kg/day? Question 2. Will you be interested in implementing and managing Low Protein Diet (LPD) for conservative management of CKD patients? Question 3. How do you suggest nephrologists can help implement more effectively Low Protein Diet (LPD) protocols for management of CKD patients?

## Supplemented protein restriction: can we have It both ways?

An effective strategy to enhance the salutary effects of a LPD is to assure that at least 50 % of the daily protein intake is of a HBV protein source such as dairy products, while the rest can be vegetarian (see Table 1). Another important consideration is provision of adequate energy along with the LPD, i.e., 30 to 35 Cal/kg/day [6]. Whereas a LPD can be implemented by adhering to dietary restriction, disease-specific, hypercaloric oral supplements may enhance the therapy without causing malnutrition. This can be pursued by supplementing daily food intake with substitutes that are manufactured to assure HBV and adequate energy intake. Some practitioners may be aware of certain commercial products used for non-dialysis CKD management, including a commercial product known as Suplena® (Abbott Nutrition Abbott Laboratories, Columbus OH, also known as Nepro LP® in some other countries [LP stands for Low Protein], not to be confused with Nepro HP® or simply Nepro® [Abbott Nutrition Abbott Laboratories, Columbus OH] in the USA [HP stands for High Protein]), a product recommended for patients with Stage 5 CKD. These and other commercially available products are designed for tube or oral feeding as supplemental or sole-source nutrition, and under medical supervision. They often have lower protein content than products designed for dialysis patients where the latter two products are still richer in protein and calories, they have less potassium, phosphorus and sodium density than non-CKD specific products (see Table 4 for comparison of some commercially available supplements in the US). There are very few studies that have examined the use of such oral nutritional supplements in non-dialysis dependent patients with CKD. In a Spanish study of 22 patients with CKD receiving a LPD (0.6 g/kg/day), half of patients also received a portion of their prescribed dietary protein and calories via Suplena® for 6 months [25]. In the oral supplement group the nutritional measures were better, while their protein intake appeared to be closer to the target LPD objective. They also had better adherence with the therapy and had greater preservation of renal function than the control group [25].

**Table 4:** Comparisons among the nutrient values of some commercially available supplements in the USA, manufactured and distributed by Abbott Nutrition or Nestle Nutrition. Information adapted from www.abbottnutrition.com, www.nestlehealthscience.us/brands, and also from Rattanasompattikul et al. [32])

	Suplena® (Nepro LP^TN^)	Nepro® (Nepro HP^TN^)	Ensure® (Original Ensure)	Renalcal®	Novasource® Renal
Volume, ml	237	237	237	250	237
Osmolality, mOsm/kg	780	745	630	600	800
Energy, Cal	425	425	220	500	475
Cal/mL	1.8	1.8	0.9	2.0	2.0
Volume, ml	237	237	237	250	237
Protein, g	10.6	19.1	9	8.5	21.6
% Calorie from Protein	10 %	18 %	16 %	7 %	18 %
Fat, g	22.7	22.7	6	20.5	23.8
Saturated Fat, g	2	2	5		
Trans Fat, g: 0.0	0	0	0		
Polyunsaturated Fat, g	4.1	4.1	2		
Monounsaturated Fat, g	16.1	16	3		
Cholesterol, mg	5.8	6.5	<2		
Carbohydrate, g	46.4	37.9	32	73	43.5
% Calorie from Carb.	44 %	36 %	58 %	58 %	37 %
Dietary Fiber, g	3	3	<1		
Sugars, g	14.8	8.4	15		
Glycerine, g	2.6	2.6	-		
Electrolytes and Minerals:					
Sodium, mg	190	250	200	15	225
Potassium, mg	270	250	370	20	225
Calcium, mg	250	250	300	15	200
Phosphorus, mg	170	170	250	25	195
Magnesium, mg	50	50	100	5	47
Iodine, mcg	38	38	38		36
Manganese, mg	0.5	0.5	1.2		1
Copper, mg	0.5	0.5	0.5		0.5
Zinc, mg	6.4	6.4	3.7	3.5	
Iron, mg	4.5	4.5	4.5		4.3
Selenium, mcg	18	18	18	12.5	26
Chromium, mcg	30	30	30		28.6
Molybdenum, mcg	19	19	38		17.9
Vitamins and Others:					
L-Carnitine, mg	63	63	-	25	62.6
Taurine, mg	38	38	-	25	35.7
Energy, Calorie	425	425	220	500	475
Vitamin A, IU	750	750	1250		712
Vitamin D, IU	20	20	200		95
Vitamin E, IU	23	23	7.5		7.2
Vitamin K, mcg	20	20	20		19
Vitamin B6, mg	2.0	2.0		1.8	
Vitamin B12, mcg	2.3	2.3		1.5	
Vitamin C, mg	25	25	30	15	14.3
Folic Acid, mcg	250	250	100	150	
Thiamin, mg	0.56	0.56		0.4	
Riboflavin, mg	0.64	0.64		0.4	
Niacin, mg	7.5	7.5		5	
Biotin, mcg	120	120		75	
Pantothenic acid, mg	3.8	3.8		2.5	

## Keto-analogues and amino acids in North America

Currently there are no commercially manufactured keto acid analogues (KA) of amino acids in the US or Canada, while there are some products with essential amino acids [26]. Shah et al. recently published a comprehensive review article on the use of KA in various countries, including the US [26]. As to why there are no KAs available in US or Canada, there may be some historical reasons, in particular the non-conclusive results of the MDRD Study [26]. Indeed, in the MDRD study, the KAs used for Study B (the VLPD supplemented with KAs) were manufactured in the US in the 1980’s and early 1990’s by Ross Laboratories (Columbus, OH), which later became Abbott Nutrition Abbott Laboratories. It can be speculated that decisions not to pursue commercialization of KAs in the US based upon the negative results of the MDRD study. It appears that the European company Fresenius Kabi (Ketosteril®, Bad Homburg, Germany) took over the product and initiated manufacturing and distribution of some types of KAs outside of the US, including South East Asia, Europe, and some Latin American countries such as Mexico. [27] In most of these countries, KAs are regulated as drugs and are relatively expensive, while in some other countries they are classified as dietary supplements. In some countries such as India, several companies manufacture and distribute KAs with some subtle differences (see Tables in article by Shah et al. [26] for comparison). It is unclear as to why these products have not yet entered the American market some 20 years after the MDRD study. It is possible that the US Food and Drug Administration (FDA) would stipulate due diligence procedures including large scale randomized controlled trials in order to approve their use in the US, if KAs were to be purposed as drugs. Alternatively, these products could theoretically enter the US market as dietary supplements or medical foods, which require a less rigid regulatory pathway and would likely be less expensive. However, approval as a dietary supplement may confound the commercial interests of the parent companies holding the patents to the use of KAs for treating CKD.

Whereas there remain many unanswered questions pertaining to the business and marketing of KAs in North America, their biological impact is a separate topic which deserves review beyond the scope of this article and is discussed elsewhere [28]. In summary, use of KAs was tested in the MDRD study, where supplementation of a VLPD with KAs and amino acids was provided in Study 2. While the results of the MDRD study and its secondary analyses did not suggest a clear benefit from this strategy, it is possible that the type of supplementation used in the MDRD study was not ideal because the KA supplement contained excessive amounts of tryptophan which could have led to production of nephrotoxic metabolites [29]. Therefore, it is possible that alternative supplementation regimens could be more beneficial to patients with CKD.

## Conclusions and dietary recommenations

The practice of nutritional management of non-dialysis CKD patients in North America appears to be severely hampered by the fear of PEW, which is a powerful predictor of outcomes in the entire range of CKD that can be alleviated by assuring adequate protein and energy intake. Many American nephrologists regard MDRD as an entirely negative study and shy away from LPD. Conversely, uncontrolled high protein intake can have deleterious consequences including biochemical imbalances such as hyperkalemia and hyperphosphatemia, and also worsening metabolic acidosis, oxidative stress, altered endothelial function, nitric oxide production, insulin resistance, and glomerular hyperfiltration leading to worsening proteinuria and uremia. Manipulation of protein intake may result in significant clinical benefits that could range from preservation of kidney function and nutritional status to improvement in PEW and alleviation of uremia. A means to that end is restricting dietary protein intake and supplementing diet with HBV protein and essential nutrients and supplements that are specifically designed for CKD patients and help deter metabolic complications, reinforced by measures that prevent the deleterious side effects of regular meals, e.g. phosphorus and potassium binder medications, a low salt diet, restricted fluid intake, and low-glycemic nutrients for diabetic CKD patients without burnt-out diabetes [30]. Whereas these dietary approaches may pose the challenge of adherence in the context of high protein diet that prevails the American mentality, they are conceptually feasible towards achieving the same goal. Registered dietitian support may improve adherence to LPD.

Our dietary recommendations for non-dialysis CKD patients with eGFR <45 ml/min/1.73 m^2^ (Stages 3b, 4 and 5, including gradually failing kidney transplants) or proteinuria >0.5 g/day include a LPD (0.6 to 0.8 g/kg/day) containing HBV proteins and/or amino acids and adequate energy intake of 30–35 Cal/kg/day. Vegetarian or alkali types of foods can be considered along with HBV proteins or commercial supplements. Diabetic CKD patients should also adhere to glycemic and calorie recommendations according to pertinent guidelines if possible although worsening hypoglycemia should be watched closely. For non-adherent CKD patients with dietary protein intake >0.8 g/kg/day based on urinary urea nitrogen estimates of the 24-h urine collections or in those with signs of PEW, we suggest the provision of oral nutritional supplements that are specifically designed for CKD patients. Patients with severe PEW or episodes of superimposed AKI may need higher protein intake (e.g. 1.2 g/kg/day or even higher, especially if hypercatabolic and in critical condition) during the critical period or temporary dialysis treatment [31]. We cautiously favor the expansion of the LPD practice in North America and suggest additional studies using these and other emerging therapies in non-dialysis dependent-CKD. As an appendix to this article we have included some example of LPD instructions and recipes for CKD patients, as posted by the NKF of the USA website: https://www.kidney.org/atoz/content/lowprotrecipes (see Additional file 1).

## Additional file

Additional file 1: Example of Low Protein Diet instructions and recipes for American CKD patients, as posted by the National Kidney Foundation (NKF) of the USA. (DOC 46 kb)
