# Supplementary material for: North American experience with Low protein diet for Non-dialysis-dependent chronic kidney disease
Source: BMC Nephrol. 2016 Jul 19;17:90. doi: 10.1186/s12882-016-0304-9 (PMC4952055; doi:10.1186/s12882-016-0304-9)
Supplement: Additional file 1: — Example of Low Protein Diet instructions and recipes for American CKD patients, as posted by the National Kidney Foundation (NKF) of the USA. (DOC 46 kb) [file 12882_2016_304_MOESM1_ESM.doc]

**Additional file 1**

**Example of Low Protein Diet instructions and recipes for American CKD patients, as posted by the National Kidney Foundation (NKF) of the USA**

<https://www.kidney.org/atoz/content/lowprotrecipes>

# **Low-Protein Recipes**

If you recently learned that you have kidney disease, your doctor may have told you to start following a low-protein diet. You may be wondering how you will be able to adjust this new diet to your usual cooking or meal planning habits. Here are some tips.

## **Why is a low protein diet necessary?**

Protein is needed for growth, upkeep and repair of all parts of your body. Protein comes from the food you eat. When your body digests it, a waste product called urea is produced. If the kidneys are not working well, urea can build up in the bloodstream and may cause loss of appetite and fatigue. Eating a low-protein diet will reduce the workload on the kidneys so that the remaining healthy part of the kidney does not have to work so hard. There are two main sources of protein:

- Animal products (fish, poultry, eggs, meat and dairy products) are considered "high quality protein." You may need to limit dairy products because they are high in phosphorus; they may cause your blood phosphorus level to be too high.
- Vegetable products (breads, cereals, rice, pasta, dried beans) are considered "low quality protein."

You will need some of each type of protein each day.

## **How can I stretch the protein I eat?**

You can "extend" protein in recipes so that a small amount seems more satisfying.

Sandwiches

- Use thinly sliced meats - it looks like more.
- Fill out sandwiches with lettuce, alfalfa sprouts, cucumber, chopped celery, apple, parsley or water chestnuts.

Soups

- Use lower protein foods such as milk substitutes for cream soups, or rice or pasta to make soups more filling without using too much protein.

Main Dishes

- Think of vegetables and grains as the "main dish" and meat as the "side dish" or complement to your meal.
- Try kebabs, using small pieces of meat and more vegetables.
- Make fried rice with vegetables and less meat or shrimp.
- Toss together a chef's salad using crisp vegetables and small strips of meat and egg.
- When making casseroles, decrease the amount of meat; increase the starch, pasta or rice and use low sodium soups when the recipe calls for soup.
- Add low-protein pastas and breads to keep protein within limits.
- Use stronger-tasting cheeses such as sharp cheddar, parmesan or romano - you'll need much less to get the same amount of flavor.

## **Calorie Boosters**

When you lower the amount of protein in your diet, you may also find the calories are lower. It is especially important to get enough calories to maintain a healthy weight at this time. In order to make up those extra calories, try some of these suggestions:

- Increase heart-healthy fats: polyunsaturated vegetable oils (made with corn, cottonseed, safflower, soybean or sunflower oils), olive oil, mayonnaise-type salad dressings.
- Use candy and sweeteners: hard candy, gum drops, jelly beans, marshmallows, honey, jam and jelly, and sugar (if you are diabetic, consult your dietitian).
- Use canned or frozen fruits in heavy syrup.

## **Modifying Recipes to Lower Protein**

Here are some examples of how you can take a typical recipe and modify it to lower the protein content:

| Festive Turkey Salad | |
| --- | --- |
| (Original Recipe) | (Modified Recipe) |
| 3 cups chopped cooked turkey breast without skin 1/4 cup diced celery 1 cup raw red delicious apples with skin 1/4 cup coarsely chopped pecans 3 tbs. low calorie mayonnaise  (Cranberry French Dressing) 1/4 cup jellied cranberry sauce 1/8 tsp. salt 1/8 tsp. paprika 1/8 tsp. dry mustard 1/8 tsp. pepper 1 tbs. vinegar 2 tbs. vegetable oil  Yield: 4 one-cup servings with 2 tbs. dressing on each serving | 1 1/2 cups chopped cooked turkey breast without skin 1 cup diced celery 3 cups raw red delicious apples with skin 1/4 cup coarsely chopped pecans 3 tbs. regular mayonnaise  (Cranberry French Dressing) 1/2 cup jellied cranberry sauce 1/8 tsp. paprika 1/8 tsp. dry mustard 1/8 tsp. pepper 1 tbs. vinegar 2 tbs. vegetable oil  Yield: 6 one-cup servings with 2 tbs. dressing on each serving |
| Combine first five ingredients in large bowl. Stir well. Cover and chill thoroughly. Serve with Cranberry French Dressing. Dressing: Combine first four dressing ingredients in small bowl, stirring with a wire wisk until smooth. Gradually add vinegar to cranberry mixture, alternately with oil, beginning and ending with vinegar. Stir well with each addition. | |

| National Renal Diet Exchanges: (per serving) | | | |
| --- | --- | --- | --- |
| Original Recipe | | Modified Recipe | |
| Meats | 6 | Meats | 1 |
| Vegetables | 1 | Vegetables | 1 |
| High Calorie | 1 | Fats | 2 |
|  |  | Fruits | 1 |
| Protein | 43 grams |  |  |
|  |  | Protein | 9 grams |
|  | | | |

Adapted from a recipe developed by the Modification of Diet in Renal Disease (MDRD) Study - University of Iowa Center.

| Chicken Pasta Casserole | |
| --- | --- |
| (Original Recipe) | (Modified Recipe) |
| 1 12-oz package egg noodles 1-1/2 lb ground chicken or beef 1 tsp. onion salt 1 tsp. garlic salt Accent to taste 1 tsp. black pepper 1 6-oz can tomato paste 1 4-oz can mushroom stems and pieces (drained) 1 8-oz container sour cream 1/4 cup cottage cheese 1/2 lb grated cheddar cheese | 1 12-oz pkg. regular or low-protein noodles 1/2 lb ground chicken or beef 1 cup diced celery 1 tsp. onion powder 1 tsp. garlic powder 1 tsp. black pepper 1 6-oz can tomato paste 1 4-oz can mushroom stems and pieces (drained and rinsed) 4 oz sour cream 3 tbs. cottage cheese 1/4 lb grated sharp cheddar cheese |
| Yields: 8 servings (cut 9" x 13" pan 4" x 2") | |
| Prepare noodles according to package directions. Drain in colander, rinse with cold water and set aside. Brown chicken (and sauté celery until tender in modified recipe). Add seasonings, tomato paste, one tomato-paste can of water and mushrooms. Place noodles in large bowl; mix in sour cream and cottage cheese. Line bottom of 9" x 13" baking dish with noodle mixture. Top with ground chicken mixture. Sprinkle cheddar cheese on top. Bake at 350, until cheese melts and casserole is heated through. | |

| National Renal Diet Exchanges: (per serving) | | | |
| --- | --- | --- | --- |
|  | Original Recipe | Modified - Reg. Pasta | Modified - Low Protein Pasta |
| Meats | 4 | 2 | 2 |
| Starch | 2 | 2 | 0* |
| Vegetables | 1 | 1 | 1 |
| Fats | 2 | 1 | 2 |
| Protein | 33 grams | 19 grams | 15 grams |
|  |  |  | (* High Calorie - 2) |
